# The Story of the Hospitals

**Published:** 1887-01-29

**Authors:** 


### THE STORY OF THE HOSPITALS.

### THE BRITISH HOME FOR INCURABLES.

(*Continued from page 245.*)

*The Dark Side: "Day and Night," "Night and Day."*

IN a former article the bright side of this Home was presented to the reader, but there is a darker corner in the picture. Some of the patients never get as far as the pleasant sitting-rooms, and think themselves lucky if now and again they are moved from the bed to an easy chair, or can find a listener to whom their defective enunciation is no bar to a brief conversation. Yet even here there is little or no complaint; nothing, indeed, but thankfulness for the care and attention they receive, and a sweet resignation which it would be impossible to try to describe. Only in one case was there a note of weariness heard; and this was an old lady who, in addition to severe paralysis, was a martyr to the acutest rheumatism. "Day and night, night and day," moaned the poor sufferer, "I pray that relief may not be long delayed." The male inmates have, as may be expected, less variety of occupation, but needle-work and some primitive wood-carving while away the lagging hours of some of them. In nearly every case they are great readers, and take a lively interest in the affairs of the great world. The Irish question has penetrated even into this home of suffering, and one gentleman, after the usual salutations were over, expressed to us his great gratification at the despatch of Sir Redvers Buller to the "distressful country." Another is a member of the Primrose League, and at the head of his bed are portraits of Lord Beaconsfield—the frame decked with the symbolical flower—and Lord Salisbury.

*Politicians: not Wilkie's.*

He told us he was expecting a picture of Lord Randolph Churchill, with an air as if he would have us understand that then his gallery of statesmen would be complete. This, however, was long before recent events. The male inhabitants of this suburban retreat have a cosy little smoking-room opening on to the garden, but, extraordinary as it may appear, there are very few devotees of the soothing weed in the Home at any time. Just now there is only one solitary smoker, and the pleasure that lights up his face when some kind friends sits down to join him in a pipe can be better imagined than described.

In addition to the good work which is done within the walls of the Home at Clapham, an even wider field of charity is covered by a most excellent system of pensions of £20 a year to out-patients. The number of the recipients of this charity is limited only by the funds at the disposal of the Committee. Last year there were 265 on the books, each of whom is under the charge of some generous and kindly "Associate" of the Home. This system of honorary lady associates is distinctive of the institution. They are to be found in every part of Great Britain and Ireland, and the nature of their duties and privileges cannot be better explained than by the following extract from the rules drawn up for their guidance by the Committee:—

"To carefully examine into, when required, and report to the Board the various circumstances of applicants to be placed on the list of candidates.

"To visit from time to time the pensioners and candidates who live in their districts, when required.

"To collect subscriptions and donations on behalf of the charity, and to assist in every way the furtherance of the work of the institution.

"In consideration of the duties entailed by appointment, all honorary lady associates or agents shall be governors of the charity, with two votes at each election, so long as they remain in office, and shall have for every £5 5*s*. remitted in any one year to 31st December, one vote at each election during the subsequent year, except for money received from any public undertaking."

*The War Chart and the Red Flag.*

Without some such assistance as this it would be almost impossible to work the out-patient department with any success, so widely distributed are the recipients. In Mr. Salmond's office in Cheapside a number of county maps cover the walls, and all over these maps are dotted little red flags. This is the "war chart" of the British Home for Incurables, every flag representing a pensioner; and reports as to the progress of these disabled ones of life's army are received at stated intervals from the lady associates. Some of the documents are most pathetic reading—living sermons on unselfishness and resignation, even under crushing disasters. One lady reports of an old man who is a hopeless paralytic, even his articulation being affected: "He has become quite like an old friend during the last nine months. Never one word of complaint. All is peace and thanksgiving, though he is unable to move a limb. He never fails to remember the busy workers in the world from which he is shut out. He usually greets me with tears of happiness, and it is not to be wondered at that I take a great interest in my work." Here is another extract from the report of a lady associate at Burton-on-Trent: "I visited Mrs. W. on February 6th. After a most pleasant talk with her, during which I told her of my interest in them, she asked me to see her husband. A more deserving case could scarcely be imagined. Paralysed in every limb, his speech at times unintelligible, he lies helpless, not able to put out his hand to greet a visitor. For five years he has not been out of doors. We soon became friends, and although he was kind enough to say, after I had sat for three-quarters of an hour with him, that it had been like an angel's visit, it was he who deserved that title and not myself, for a more sweet example of Christian trust I have never seen. This happiness under every circumstance calculated to distress, is most wonderful. If the contributors to the Home could know how great a privilege it is to alleviate the sufferings of such a case they would be glad, as I am, to belong to it."

Saved from the Wreck.

These are only samples of many a score of documents of a like kind—all of which tell of patient suffering, of heroic self-sacrifice on the part of husbands, wives, brothers, and sisters of the afflicted, and of gratitude beyond expression to the Home for the assistance it renders, and to the lady associates for their sympathy, which is even more precious than gold and silver. These Christian gentlewomen might say, without flattering themselves, as they notice the brightness and joy which steals over the faces of their charges as they enter the room: "A wreck past hope he was; his life I gave him, and did add thereto my love."

By way of concluding this long narrative, let us say that there is a special fund from which unsuccessful candidates get some small relief, and that in those years, when the finances admit, the longest unsuccessful applicant for a pension comes in without election. And now a word as to financial matters. The out-pension department disburses some £5,300 a year, whilst the annual cost of the Home is close upon £3,000. In regard to this latter item, an idea may be formed of the careful supervision which is exercised over the expenditure when it is stated that the management charges, as against the cost of maintenance, are only seven per cent. Two inmates and thirteen out-pensioners are paid for by the Mitchell Charity—an old City trust for which, to this extent, the Home acts as the machinery for dispensing its gifts. As an instance of the enormous need there is for an institution such as we have attempted to describe, it may be added that from 600 to 700 applications from incurables seeking to participate in its benefits are received every year. Careful selection reduces this total to about 100, of whom, however, only twenty can be elected. To reduce the number of these disappointed ones is an endeavour in which everyone may well be glad to take some part, however humble.

Ways and Means.

